# Genetic Determinants of Cell Size at Birth and Their Impact on Cell Cycle Progression in *Saccharomyces cerevisiae*

**DOI:** 10.1534/g3.113.007062

**Published:** 2013-09-01

**Authors:** Sandra K. Truong, Ryan F. McCormick, Michael Polymenis

**Affiliations:** Department of Biochemistry and Biophysics, Texas A&M University, College Station, Texas 77843

**Keywords:** START, channelyzer, G1 phase

## Abstract

In most cases, cells must increase their size before they can divide. Hence, a small size has been used often as a phenotype for mutants that accelerate initiation of division, such as the celebrated *WHI* mutants of budding yeast. Recently, we measured the DNA content of all nonessential gene deletion strains in *Saccharomyces cerevisiae*. Surprisingly, there was little, if any, correlation between mean cell size and cell-cycle progression. Here, we examine this issue further, providing the first systematic analysis of genetic determinants of the cell size at birth. We found that although a large birth size strongly correlates with a large mean size, the converse relationship (*i.e.*, small birth size *vs.* small mean size) is not as strong. Our data also suggest that mutants that are born large do not have a significant advantage for faster cell-cycle progression. In contrast, mutants that are born small are more likely to progress slower in the cell cycle. The majority of gene deletions that displayed such phenotypes affect protein synthesis or ribosome biogenesis. Overall, our data suggest that birth size may be a more informative parameter for cell-cycle progression than the mean size of a proliferating cell population. In contrast to *WHI* phenotype expectations, a small size is more likely to be associated with delayed cell-cycle progression.

The amount of cytoplasm per nuclear DNA represents one of the strongest allometric relationships among cells of different species or among ploidy variants of the same species (Jorgensen and Tyers 2004; Turner *et al.* 2012). However, to what extent genetic determinants of overall size homeostasis affect the DNA content of proliferating cells is not clear. We previously used flow cytometry to measure the DNA content of all *Saccharomyces cerevisiae* nonessential gene deletions (Hoose *et al.* 2012). Contrary to expectations, we reported very little correlation between the DNA content of mutant strains and the mean cell size of these mutants (Hoose *et al.* 2012). Subsequent work by others confirmed these observations (Dungrawala *et al.* 2012). These results argued that genetic determinants of overall size homeostasis are neither the sole nor the main factor determining cell-cycle progression and the timing of initiation of cell division in proliferating cells. However, mean cell size of a population is a complex ensemble of cell size at birth, at initiation of division, or at some other cell-cycle point (for an excellent recent review on cell size control, see Turner *et al.* 2012). To illustrate such complexity, some mutants with small mean size (*e.g.*, *sfp1Δ* cells) may have a small birth size and a reduced critical threshold for initiation of division (Hoose *et al.* 2012). Alternatively, other small-size mutants (*e.g.*, *rps0bΔ* cells) are also born small but have an increased critical size threshold for initiation of division (Hoose *et al.* 2012). To dissect the relationship of cell size and cell-cycle progression further, we focused specifically on cell size at birth. Surprisingly, the extent that birth size determines mean size depends on whether cells are born small or large. It is much more likely for cells that are born large to stay large than it is for small cells to stay small. We also found that cell-cycle progression is more correlated with birth size than with mean size.

## Materials and Methods

### Data sources and acquisition

**Figure 1:**
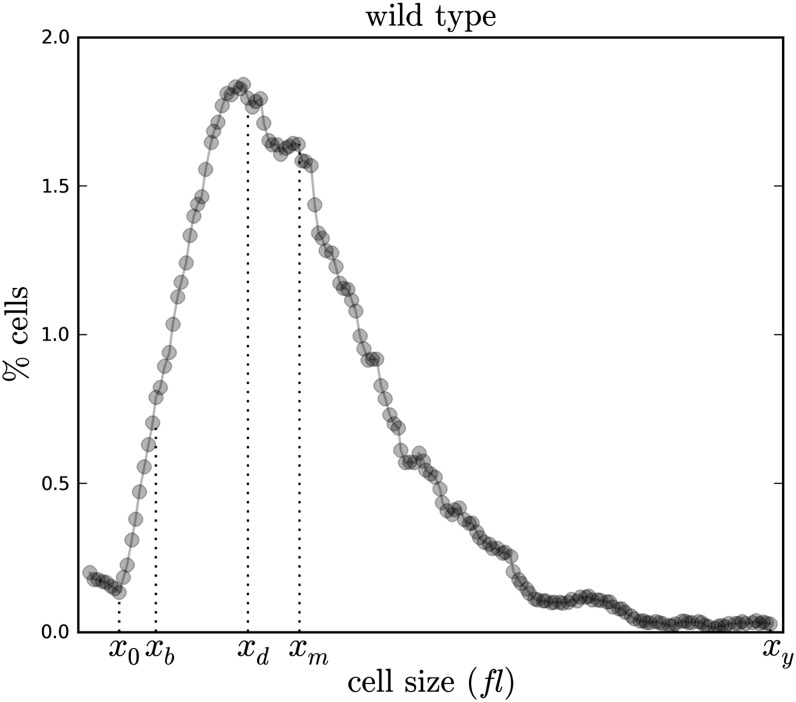
Illustration of cell size distribution parameters. For this analysis, five parameters, *x_0_*, *x_b_*, *x_d_*, *x_m_*, and *x_y_*, were determined for cell size distributions obtained from Jorgensen *et al.* (2002). The size distribution shown is from a wild-type sample shown in Jorgensen *et al.* (2002). For a given cell size distribution, *x_0_* represents the start of the distribution and was determined visually from the distribution by excluding experimental noise. *x_d_* represents maximum daughter cell size and is a visual approximation of the mode of the cell size distribution. *x_b_* represents the maximum birth size and is approximated by the maximum of a proportion of the daughter cell interval. *x_m_* represents the mean cell size of the distribution. *x_y_* represents the end of the distribution and was determined as the last cell size in the distribution. Once parameters were determined, we calculated Spearman’s rank correlation coefficients between: *x_b_* and *x_m_*, *x_b_* and %G1, and *x_b_* and fitness for four categories of mutants (see Figure 2 for illustration of category determination). %G1 and fitness data were obtained from Hoose *et al.* (2012).

The data used for the analysis of yeast birth size is shown in the Supporting Information, File S1. The cell size data were obtained from (Jorgensen *et al.* 2002), represented as a heat map in Figure 1B of that paper. For all the ORFs we analyzed here this information can also be found in the sheet labeled as “asynchronous” in File S1. The fitness and %G1 data are available in the supporting information of Hoose *et al.* (2012).

### Methods to obtain cell size distribution parameters and birth size categories

**Figure 2:**
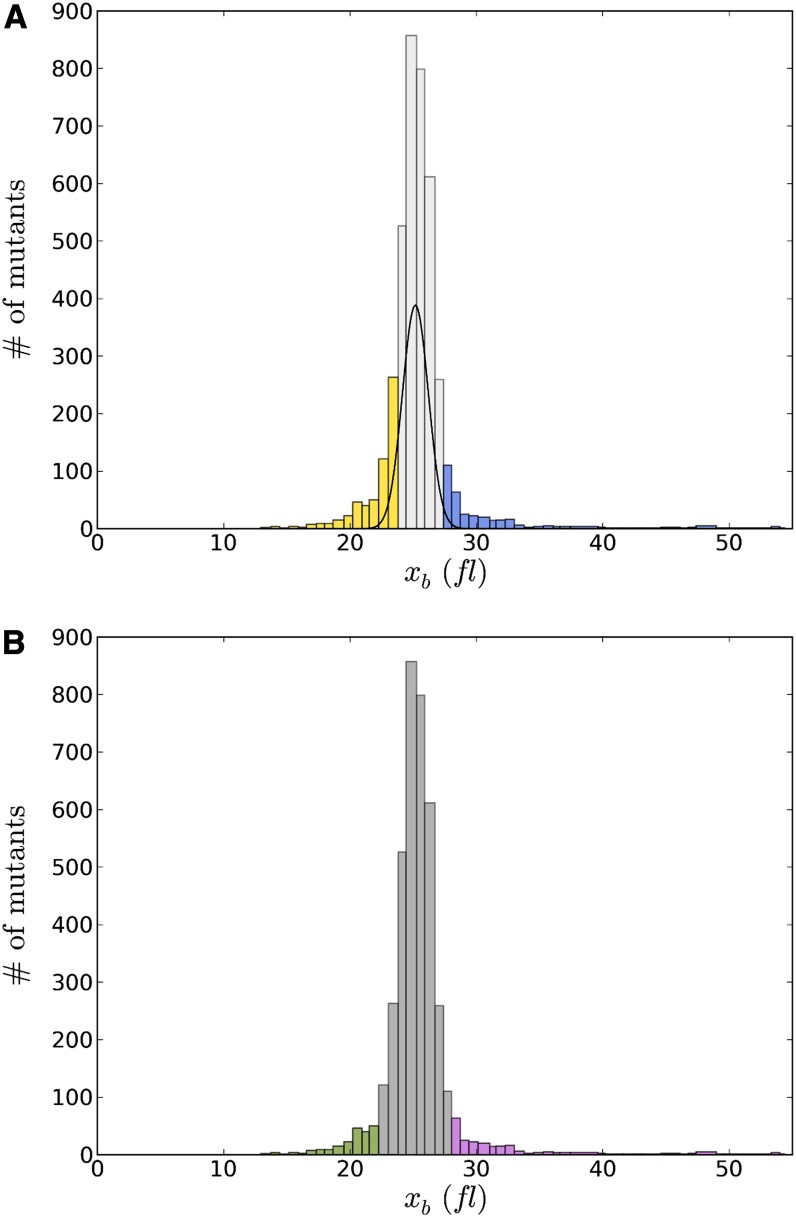
Cell size category determination. (A) Categories determined by deviations from wild-type mean *x_b_* (birth size). After calculating the mean and SD of *x_b_* for wild-type strains, mutants binned with *x_b_* less than two SDs below the wild-type mean *x_b_* were categorized as small (yellow); mutants binned with *x_b_* within two SDs of the wild-type mean *x_b_* were categorized as normal (light gray); and mutants binned with *x_b_* greater than two SDs above the wild-type mean *x_b_* were categorized as large (blue). The final category, overall, was comprised of all mutants. A normal distribution approximating wild-type *x_b_* was scaled and superimposed over the distribution of mutant *x_b_* values. (B) Categories determined as a proportion of all *x_b_*. Mutants in bins containing the smallest 5% *x_b_* were categorized as small (green); mutants in bins containing the largest 5% *x_b_* were categorized as large (purple); and mutants in bins that were not in small or large bins were categorized as normal (dark gray). The final category, overall, was comprised of all mutants.

For each strain, the five cell size parameters, *x_0_*, *x_b_*, *x_d_*, *x_m_*, and *x_y_*, were measured from cell size distributions (Figure 1) obtained from Jorgensen *et al.* (2002). Birth size categories were defined by the use of calculated *x_b_* values (Figure 2). The methods used to obtain parameters work under the assumption that a cell size distribution can be described by a continuous function. However, in practice, cell size data are discretely binned by the Coulter Channelyzer during acquisition. That is, every data point within a bin has an identical value; cell sizes within a bin cannot be distinguished. Furthermore, the intervals between bins are not identical, ranging from 0.385662112 to 1.203735813 fl. As such, the precision of our analysis is limited by this binning; any given parameter value is subject to the error *x_p_*± (*x_p_*_+1_ − *x_p_*), where *x_p_* is the cell size bin for a given parameter. The size distribution data for all ORFs can be found in the sheet labeled as “asynchronous” in File S1.

### Cell size distribution parameters

#### Filtering noise by defining x_0_ and x_y_:

Cell size distributions vary between mutants. However, a substantial interval exists such that the proportion of observations can be described as a concave function *f*(*x*), where the number of observations are first increasing and then decreasing. We assumed that observations in this interval are yeast cell size observations. For a particular mutant, we described the cell size distribution with a function *f*(*x*) for the frequency of cells in the population found at size *x*. The interval of asynchronous culture cell sizes in which yeast cells exists is found in $\left[x_{0},x_{y}\right]$, such that$\int_{x_{0}}^{x_{y}}f(x)dx=1$. Outside the cell size interval, $\left[x_{0},x_{y}\right]$, there are often spurious observations. We attributed those to noise and defined $x_{0}$ such that this noise was not included in the cell size interval. An example of this filtration can be observed in Figure 1 where the observations before *x*_0_ are considered noise. This cell size interval was visually inspected and manually curated for all mutants. We removed 17 samples from the mutant pool due to abnormal profiles from which it was impossible to define a daughter cell range. Two of those abnormal profiles are shown in Figure S1. Data for the remaining 3981 mutants used in this analysis are present in the File S1.

#### Separation of daughters from mothers by defining x_d_:

In a proliferating budding yeast population, at least half of all cells are newborn daughters. *Saccharomyces cerevisiae* divides asymmetrically, with daughter cells being smaller than their mothers (Hartwell and Unger 1977; Johnston *et al.* 1977). Hence, in a size distribution, Jorgensen *et al.* (2002) reasoned that daughter cells would occupy the “left-of-mode” area of a cell size histogram of asynchronously dividing cells (Figure 1). We think this is a logical approach because daughter cells are smaller than mother cells are. Here, we also separated daughters from mothers by partitioning at the most frequently observed cell size. We partitioned the yeast cell size interval $\left[x_{0},x_{y}\right]$ into daughter and mother cells by visually approximating the mode, *x_d_*, such that $x_{d}=\max_{x\in [x_{0},x_{y}]}f(x)$. Hence, the daughter cell interval is $\left[x_{0},x_{d}\right]$ and the mother cell interval is$\left[x_{d},x_{y}\right]$.

#### Approximation of birth size, $x_{b}$, using the daughter interval $\left[x_{0},x_{d}\right]$:

Given a distribution, we calculated birth size, *x_b_*, such that it is the cell size at which the summation of all observations from *x_0_* to *x_b_* is equal to 20% of observations in the daughter cell interval $\left[x_{0},x_{d}\right]$. More precisely, it was calculated to satisfy$\int_{x_{0}}^{x_{b}}f(x)dx=\frac{1}{5}\int_{x_{0}}^{x_{d}}f(x)dx$. The 20% value was chosen for all the correlation graphs we show. However, we also analyzed *x_b_* for 10%, 15%, and 25% of observations in the daughter cell interval (see Supplementary Dataset 1, sheet labeled ‘parameters’). To accommodate the binning of the data, we solved for *x_b_* such that it satisfies $\frac{1}{5}\overset{x_{d}}{\underset{x=x_{0}}{\sum }}f(x)\in [\overset{x_{b}}{\underset{x=x_{0}}{\sum }}f(x),\overset{x_{b+1}}{\underset{x=x_{0}}{\sum }}f(x))$. That is, starting with the first bin, observations were summed sequentially for each bin until the sum was greater than or equal to 20% of the number of daughter cell observations; the bin at which this threshold was reached was used as the birth size. Therefore, a limitation of our heuristic is that we have an error for birth size $x_{b}\pm (x_{b+1}-x_{b})$ for $x_{b},x_{b+1}\in [x_{0},x_{d}]$.

#### Mean size, $x_{m}$, from total cell size distribution:

The mean size, *x_m_*, is the average cell size observed. That is, $x_{m}=\sum_{x=x_{0}}^{x_{y}}x\;f(x)$. Recall that *f*(*x*) describes the frequency of cells in the population found at size *x*, $\int_{x_{0}}^{x_{y}}f(x)dx=1$, and thatf(x)∈[0,1]. Once again, the precision is dictated by the bin range. Hence, there is an error for mean size where $x_{m}\pm (x_{m+1}-x_{m})$.

### Birth size categories

In the past, Hoose *et al.* (2012) used deviations from wild-type mean to identify mutant DNA content outliers, whereas Jorgensen *et al.* (2002) used 5% cutoffs relative to all mutant profiles to identify small and large mutants. Hence, we decided to use both of these methods to generate categories of mutants with respect to their calculated birth size, *x_b_*. Although there is overlap for most mutant outliers (small and large), the cutoffs are different (see Figure 2).

#### Categories with respect to wild type:

In the first method, categories were determined by deviations from wild type mean *x_b_* (birth size). After we calculated the mean and SD of *x_b_* for wild-type strains, mutants binned with *x_b_* less than two SDs below the wild-type mean *x_b_* were categorized as small; mutants binned with *x_b_* within two SDs of the wild-type mean *x_b_* were categorized as normal; and mutants binned *x_b_* greater than two SDs above the wild type mean *x_b_* were categorized as large. The final category, overall, comprised all mutants.

#### Categories with respect to 5% cutoffs of all mutants’ x_b_:

In the second method, categories were determined as a proportion of all *x_b_*. Mutants in bins containing the smallest 5% *x_b_* were categorized as small; mutants in bins containing the largest 5% *x_b_* were categorized as large; and mutants in bins that were not small or large bins were categorized as normal. The final category, overall, was comprised of all mutants.

### Correlations with other datasets

We correlated birth size values with the mean cell size (Jorgensen *et al.* 2002), overall fitness (Giaever *et al.* 2002), and with DNA content (Hoose *et al.* 2012) of the corresponding deletion strains. All the mutants were in the s288c strain background. However, the cell size dataset was from haploids (strain BY4741; (Jorgensen *et al.* 2002)), whereas the fitness (Giaever *et al.* 2002) and DNA content (Hoose *et al.* 2012) datasets were from diploids (strain BY4743). Haploids would be more sensitive than diploids to the effects of recessive mutations acquired during strain construction or afterward. Hence, it is possible that there will be some loss in the concordance between the haploid and diploid sets. However, we chose these datasets because in all these studies cells were grown in liquid cultures, in the same rich (YPD − 1% yeast extract, 2% peptone, 2% dextrose) media (Kaiser *et al.* 1994), allowing for physiologically relevant analyses. For comparisons between datasets, statistical tests were performed using the open source software package R and the SciPy package for the open source Python language. The tests used in each case are indicated in the legend of the corresponding figure or table. Plots were made using the matplotlib package for the Python language. For Gene Ontology enrichments we used the YeastMine feature of the Saccharomyces Genome Database (http://yeastmine.yeastgenome.org/yeastmine/), with the Holm-Bonferroni multiple hypothesis test correction.

### Synchronous cell-cycle analysis

The synchronous cell-cycle profiles shown in Figure S2 were done using centrifugal elutriation as we described previously (Hoose *et al.* 2012), except that the strain we used here was W303a (*MAT*a *leu2-3,112 trp1-1 can1-100 ura3-1 ade2-1 his3-11,15*), cultured in synthetic complete medium (Kaiser *et al.* 1994) with 2% dextrose.

## Results and Discussion

### Defining birth size from asynchronous cell-size distributions

*S. cerevisiae* daughter cells are smaller than their mothers are (Hartwell and Unger 1977). Hence, in a size distribution of a proliferating population, the daughters would be expected to occupy the left-of-mode area. This approach was used previously to estimate daughter cell size (Jorgensen *et al.* 2002). We reasoned that focusing on the smallest cells from the ‘daughter area’ of size distributions one could obtain estimates of “birth” sizes (see the section *Materials and Methods* for detailed descriptions). However, deciding on the cutoff defining these small cells is arbitrary. Ideally one would like such a cutoff to be as low as possible, but clearly distinguishable from low counts due to “noise.” To identify a suitable cutoff, we decided to use the increase in protein abundance during G1 as a metric, comparing it to the increase in cell size calculated with our approaches. From highly accurate single cell analysis of daughter cell-cycle progression using protein-based markers of “size,” it was calculated that from birth to budding, there was a 27% increase in the abundance of the strongly expressed actin reporter (S. Di Talia, personal communication; and Di Talia *et al.* 2007). Using the same strain and medium, we obtained a homogeneous, unbudded daughter cell population in early G1 phase by using centrifugal elutriation (Figure S2). We then monitored over time cell size (using a channelyzer) and the percentage of budded cells (using phase microscopy). From these experiments, we found that new buds began to appear when cell size was at ~35 fl (see Figure S2). We also estimated the “birth” size from size distributions of asynchronously dividing cells (see *Materials and Methods*).

Using a 10% cutoff, from the smallest 10% of daughter cells, we found that birth size was 21.84 ± 0.37 fl. For a 25% cutoff birth size was 27.40 ± 0.78 fl. Hence, the birth-to-budding increase in cell size would be 60% when a 10% cutoff was used and 28% when a 25% cutoff was used. It would appear that a 25% cutoff would match the value of the birth-to-budding increase in cell size from the highly accurate protein based single-cell analysis (Di Talia *et al.* 2007). However, in those high-resolution studies budding was scored molecularly, from the appearance of the myosin ring at the bud site (Di Talia *et al.* 2007). In contrast, we certainly scored budding at a later point, when the bud grew to be clearly visible by phase microscopy. Therefore, we decided that a 25% cutoff in a size distribution is the highest threshold we could use for birth size estimates. For the lowest threshold, we used a 10% cutoff. We cannot exclude the possibility that some mutant strains may have highly variable birth sizes. In that case, the apparent birth size we would obtain would be larger than the size of a significant fraction of newborn daughters in that mutant. This is a limitation of our population-based methodology. Nonetheless, we also varied the cutoff between 10% and 25%. Among these different cutoffs, the results we present are in reasonable agreement (see File S1), supporting our overall approach.

### Does size at birth correlate with mean size?

**Figure 3:**
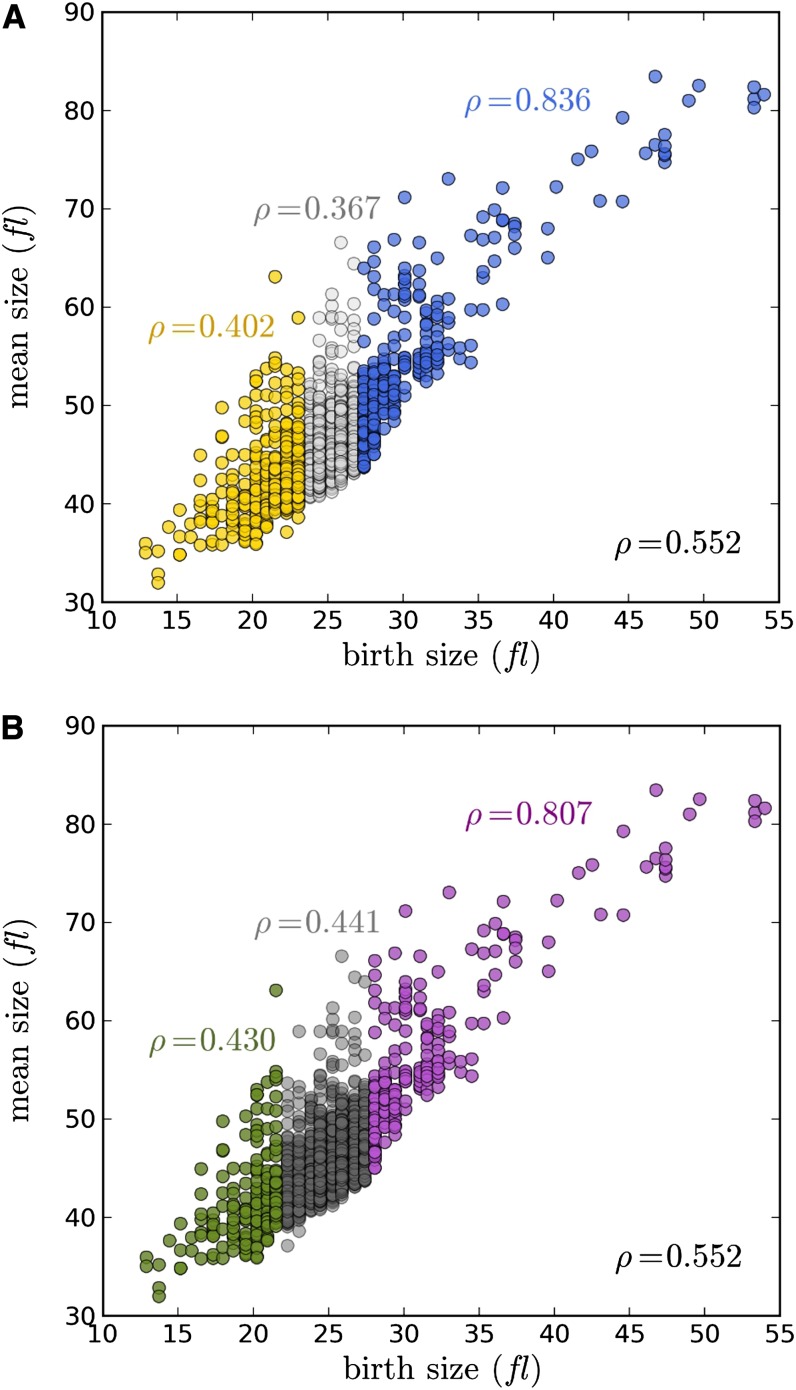
Large birth size correlates much better with mean size than small birth size does. (A) Mean size (in fl) was the average of each yeast strain’s asynchronous cell size distribution from the Jorgensen dataset (Jorgensen *et al.* 2002). The colored data represent birth size outliers (using a 20% cutoff) *μ_WT_* ± 2 SD, from the wild type (BY4741; 23.7 fl ± 0.99). In yellow are small birth size outliers and in blue large birth size outliers. All the values are in the File S1. We used the nonparametric Spearman test to obtain the correlation (*ρ*) values we show. The correlation coefficient for all the strains is shown at the bottom right of the graph. The other *ρ* values are colored similarly to their corresponding sub-groups. (B) The same analysis as in panel A, except that the categories were determined as a proportion of all *x_b_* (see *Materials and Methods*).

To what extent do genetic determinants of birth size also determine the mean size of the population? If the size(s) at which cells pass through subsequent cell-cycle transitions are affected in a manner analogous to birth size variations, then a strong correlation between birth size and mean size is expected. Indeed, this seems to be the case for mutants that are born large, since in the overwhelming majority of those mutants the mean size was also large (*ρ* = 0.83; see Figure 3). Surprisingly, however, many mutants with small birth size did *not* have a small size overall (*ρ* = 0.40; see Figure 3). Hence, “large” birth size mutants have a much stronger deterministic behavior than “small” birth size mutants do. The reasons for this different behavior are not clear.

We also noted that the “large” and “small” birth size groups were enriched for different gene ontologies. Gene ontologies classified under “cytoplasmic translation” and “ribosome biogenesis” predominate in the “small” birth size group (see File S1, sheets labeled ‘GO outliers _’). In contrast, ontologies related to chromosome organization and gene expression were prominent in the “large” birth size group (see File S1, sheets labeled ‘GO outliers _’). Therefore, different molecular pathways may impinge on birth size control, with distinct deterministic outcomes.

### Altered birth size is associated with reduced fitness

**Figure 4:**
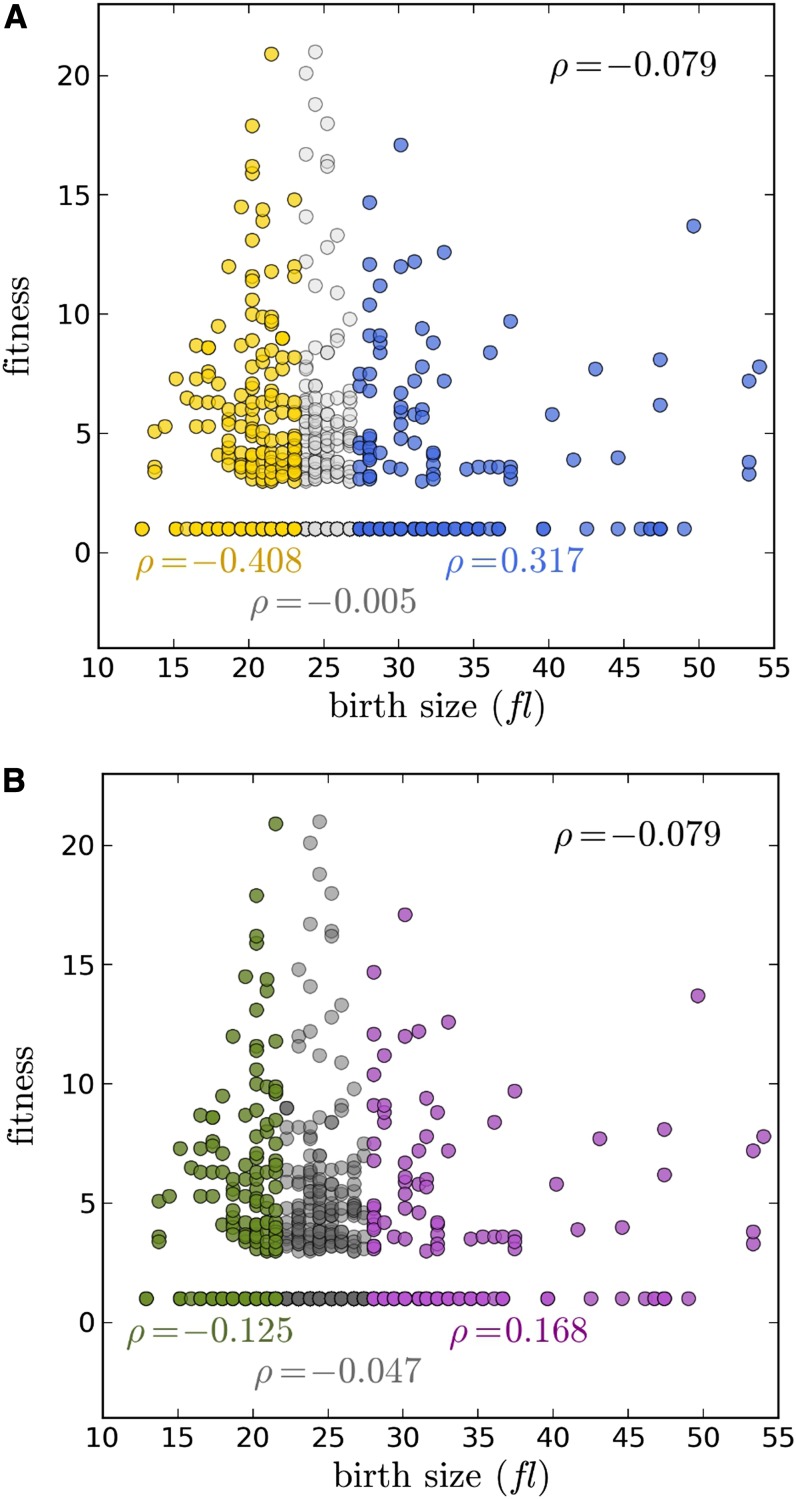
Correlation of birth size with fitness. Fitness values were obtained from the Giaever dataset (Giaever *et al.* 2002), and they range from 1 to 21, where 1 is the most fit and 21 is the least fit. In A and B, the data were colored and displayed as in Figure 3, using again the 20% cutoff for birth size estimates (see *Materials and Methods*).

We next asked whether birth size alterations are associated with a fitness penalty (Figure 4). We found that birth size mutants were more likely to have reduced fitness than mutants with normal size (Figure 4). Despite the aforementioned correlations, it is important to stress that many birth size mutants in either group did not have significant fitness defects (corresponding to “WT” fitness values of 1 in Figure 4).

### Birth size correlates better with cell-cycle progression than mean size does

**Figure 5:**
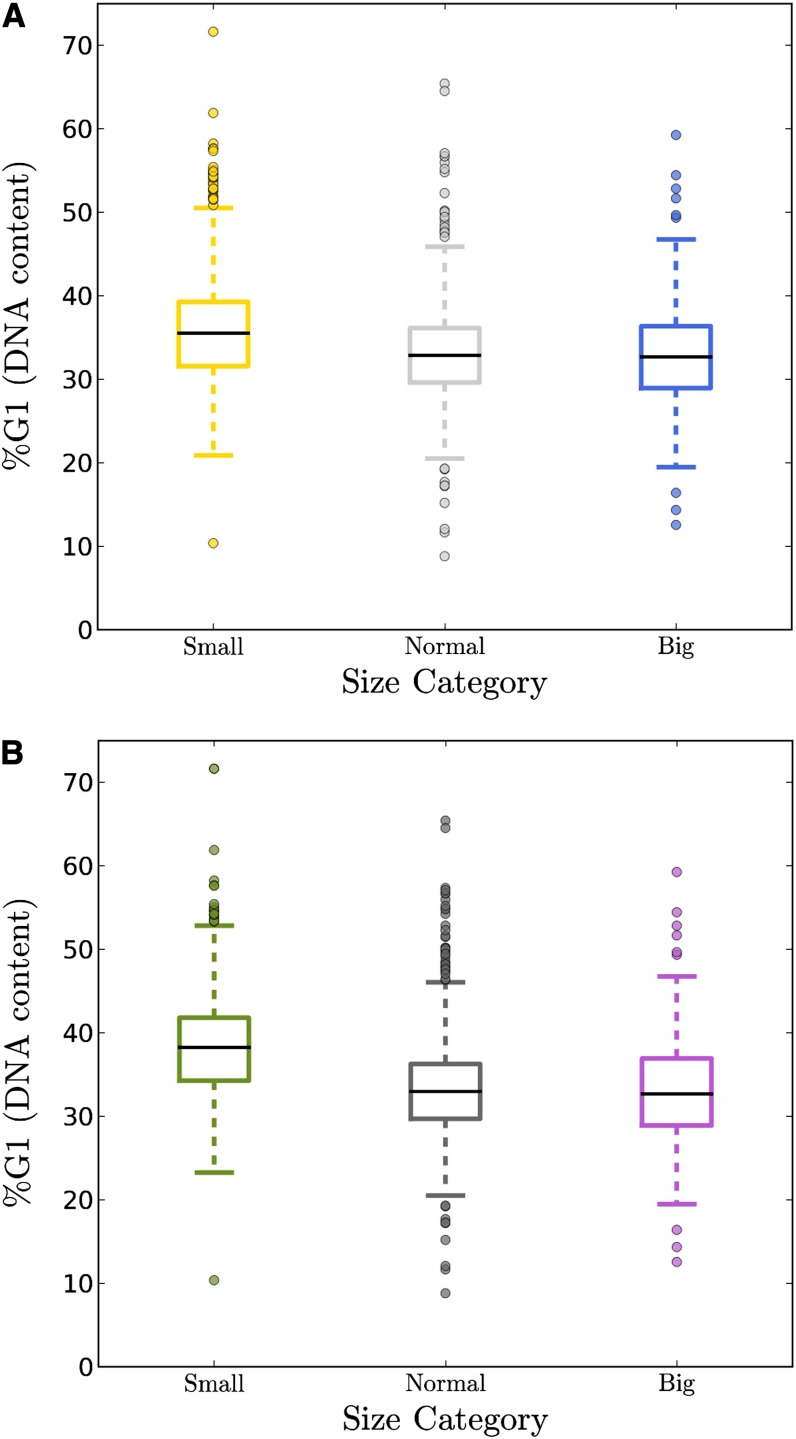
Mutants with small birth size have a higher G1 DNA content. (A) %G1 DNA content values were from the Hoose dataset (Hoose *et al.* 2012). The data were colored as in previous figures, using a 20% cutoff to estimate birth sizes (see *Materials and Methods*). The box represents the middle 50% of the data range (from the 25th percentile to the 75th percentile). The band within the box is the mean. The ends of the whiskers represent the lowest datum still within 1.5 of the interquartile range of the lower quartile, and the highest datum still within 1.5 IQR of the upper quartile. Any data points not included within the whiskers are shown as outliers, displayed as filled circles. (B) The same analysis as in panel A, except that the categories were determined as a proportion of all *x_b_* (see *Materials and Methods*).

From DNA content distributions of asynchronous cultures, the duration of the G1 phase relative to the other phases of the cell cycle can be obtained. In our previous study (Hoose *et al.* 2012), we found that there was very little correlation between mean cell size and cell cycle progression. Specifically, mutants with small or large mean size were not significantly overrepresented in the group of mutants with a high G1 DNA content (*ρ* = −0.09). Here, we observed a slightly better correlation when we correlated birth size with DNA content (*ρ* = −0.17; see Figure S3, and File S1). Interestingly, however, mutants with “large” birth size still did not display a significant overall shift in their DNA content (see Figure 5, *P* > 0.20, Table S1, and File S1). If unaccompanied by other changes, a large size at birth would lead to a relative shortening of the G1 phase. However, it appears that mutants that are born large have a compensatory delay later in G1, resulting in no net change in the overall relative length of G1. Such a putative delay is consistent with the data in Figure 3, showing that mutants that are born “large” will likely stay large. From the data we present here, it is difficult to say whether there might be contributions from cryptic cell size checkpoints later in the cell cycle, in the G2 phase.

Many mutants with small birth size also had normal %G1 DNA content (see Figure 5, and File S1). Overall, however, as a group small birth size mutants were much more likely to have a high %G1 DNA content (Figure 5). The effect was highly significant (see Table S1 for all the statistics comparing the differences between the means of each category, as shown in Figure 5, A and B). Note that the “high G1” group of mutants is also enriched for “cytoplasmic translation” and “ribosome biogenesis” gene ontologies (Hoose *et al.* 2012) as the “small” birth size group is (see File S1, sheets labeled ‘GO outliers _’). Birth size values are rarely incorporated in estimates of G1 progression. In principle, however, variations of G1 length among different mutants, or growth in different nutrients, could arise from differences in the boundaries of G1 in each case (*e.g.*, different mutants may enter and/or exit G1 at different sizes) and differences in the rate (*i.e.*, growth rate) at which cells traverse G1 in each case. Our results that small birth size correlates with G1 delay fit these predictions.

Taken together, we conclude the following in regards with the role of genetic determinants of cell size in cell-cycle progression: (1) Most size control genes have no effect on cell-cycle progression; (2) mutants that are born large do not seem to have an “advantage” for faster cell cycle progression; and (3) in contrast, mutants that are born small are more likely to be “handicapped” and progress slower in the cell cycle. Hence, of the size mutants that do affect cell-cycle progression, the majority display a small size at birth and a *delayed* initiation of DNA replication.

What are the implications of these findings, in the context of previous size-based approaches to identify START regulators? For decades, focusing on cell size alterations has been a prevalent criterion for identifying START regulators (Carter and Sudbery 1980; Sudbery *et al.* 1980; Jorgensen *et al.* 2002; Zhang *et al.* 2002). A small size at the time of initiation of division would lead to an accelerated START, but only if it is not accompanied by any other changes that prolong G1. Indeed, this is the case for some well-known mutants with truly accelerated START, such as *CLN3-1* (Cross 1988; Nash *et al.* 1988) and *whi5Δ* (Costanzo *et al.* 2004; de Bruin *et al.* 2004). However, as we have argued previously (Hoose *et al.* 2012), a small size at division in some other reported “whi” mutants was not sufficient for START acceleration. Ignoring other parameters that also affect the overall length of the G1 phase, such as birth size and growth rate, could lead to erroneous conclusions about the timing of initiation of division. Our results showing that small birth size correlates with longer G1 illustrate this problem. Overall, the emphasis on size mutants to identify mechanisms that determine the timing of initiation of cell division is problematic for two reasons: First, it could lead to errors about the actual timing of START; Second, it does not allow the sampling of gene products that do *not* affect size homeostasis. These factors represent the majority of mutants that affect initiation of cell division (see (Hoose *et al.* 2012) and this work). Hence, previous size mutant hunts have provided a limited view of the pathways that determine the timing of initiation of cell division and cell proliferation.

## Supplementary Material

Supporting Information
