# Supplementary material for: Genetic Determinants of Cell Size at Birth and Their Impact on Cell Cycle Progression in Saccharomyces cerevisiae
Source: G3 (Bethesda). 2013 Sep 1;3(9):1525–30. doi: 10.1534/g3.113.007062 (PMC3755912; doi:10.1534/g3.113.007062)
Supplement: Supporting Information [file supp_3_9_1525__index.html]

Genetic Determinants of Cell Size at Birth and Their Impact on Cell Cycle Progression in Saccharomyces cerevisiae — Supporting Information 

# Genetic Determinants of Cell Size at Birth and Their Impact on Cell Cycle Progression in *Saccharomyces cerevisiae*

## Supporting Information for Truong, McCormick, and Polymenis, 2013

**Files in this Data Supplement:**

- Supporting Information - Figures S1-S3, Table S1, and File S1 (PDF, 710 KB)
- Figure S1 - Examples of censored cell size distributions (PDF, 284 KB)
- Figure S2 - Budding as a function of size for strain W303a in synthetic complete medium (PDF, 278 KB)
- Figure S3 - Correlation of birth size with DNA content (PDF, 398 KB)
- Table S1 - Statistics of comparisons between different categories of birth size mutants and their corresponding %G1 DNA content (PDF, 243 KB)
- File S1 - Gene ontology obtained through yeastmine.yeastgenome.org for the "small" and "large" 20% birth size, x\_b, outliers defined by 5% extremes (.xls, 12 MB)
