# Supplementary material for: Genetic Determinants of Cell Size at Birth and Their Impact on Cell Cycle Progression in Saccharomyces cerevisiae
Source: G3 (Bethesda). 2013 Sep 1;3(9):1525–30. doi: 10.1534/g3.113.007062 (PMC3755912; doi:10.1534/g3.113.007062)
Supplement: Supporting Information [file supp_g3.113.007062_007062SI.pdf]

G3: Genes | Genomes | Genetics

Genetic determinants of cell size at birth and their impact on cell cycle progression in *Saccharomyces cerevisiae*

Sandra K. Truong, Ryan F. McCormick and Michael Polymenis

Department of Biochemistry and Biophysics, Texas A&M University, College Station, TX 77843

DOI: 10.1534/g3.113.007062

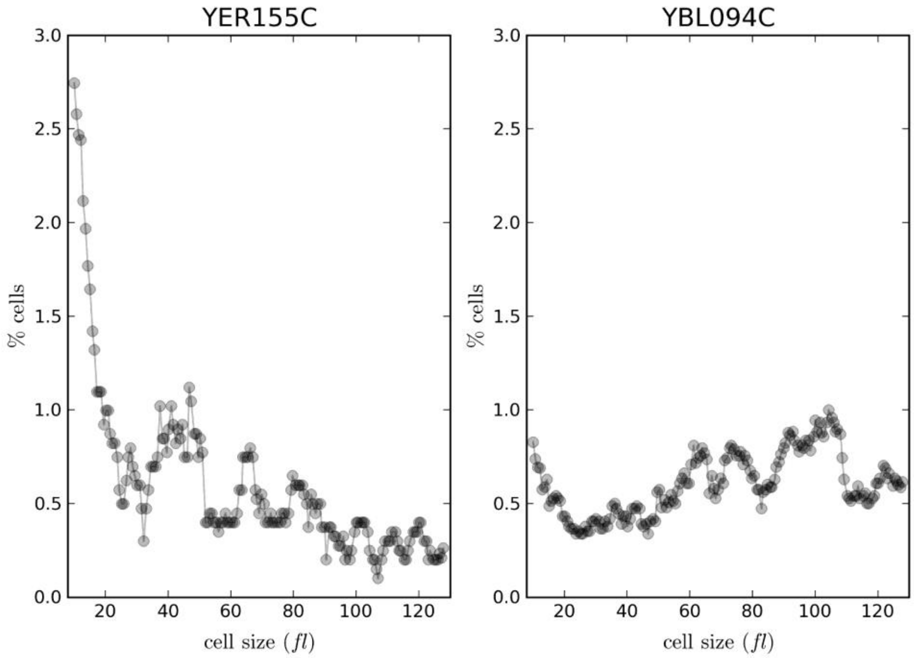

Figure S1 Examples of censored cell size distributions. These mutants' distributions were too irregular to define a daughter cell range, and they were removed from the mutant set analyzed in this paper.

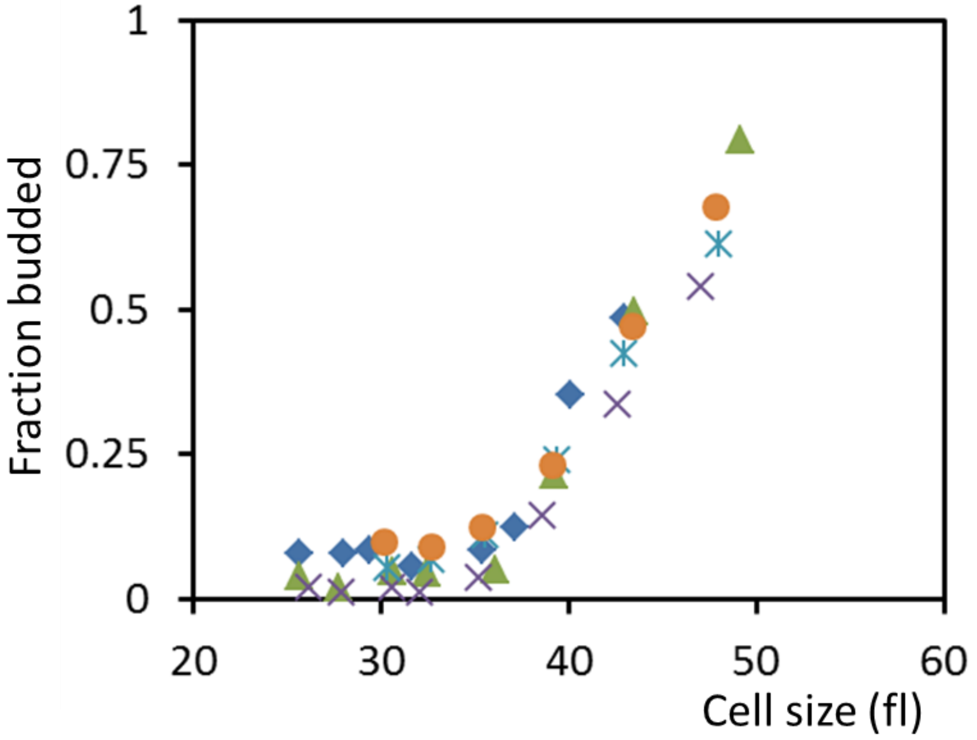

Figure S2 Budding as a function of size for strain W303a in synthetic complete medium. Cells were cultured and elutriated on five separate experiments. Every 20 min the fraction of budded cells was measured microscopically (y-axis), and plotted as a function of the mean cell size of the population (x-axis).

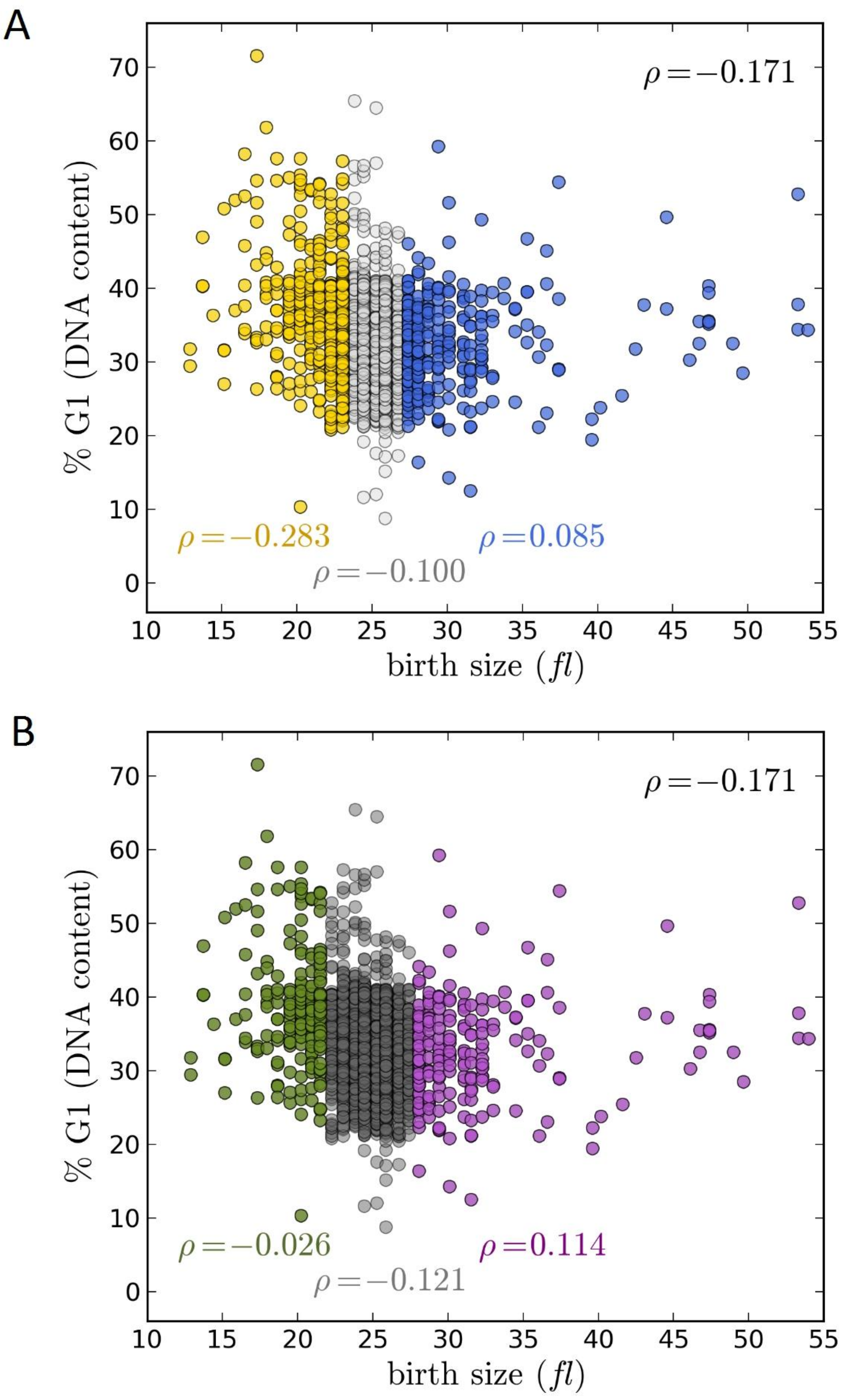

Figure S3 Correlation of birth size with DNA content. %G1 DNA content values were obtained from (HOOSE *et al.* 2012). In A and B, the data were colored and displayed as in Figure 3, using again the 20% cutoff for birth size estimates (see Materials and Methods).

File S1

Gene ontology obtained through yeastmine.yeastgenome.org for the "small" and "large" 20% birth size, x_b, outliers defined by 5% extremes

File S1 is available for download at http://www.g3journal.org/lookup/suppl/doi:10.1534/g3.113.007062/-/DC1.

Table S1 Statistics of comparisons between different categories of birth size mutants and their corresponding %G1 DNA content

Comparisons	Test	p-value
Small vs. normal birth size (see Fig. 5A)	t-test	1.3192E-37
	Mann-Whitney	5.9898E-26
Large vs. normal birth size (see Fig. 5A)	t-test	0.48
	Mann-Whitney	0.22
Small vs. normal birth size (see Fig. 5B)	t-test	2. 7059E-55
	Mann-Whitney	8. 1026E-33
Large vs. normal birth size (see Fig. 5B)	t-test	0.53
	Mann-Whitney	0.24
