# Supplementary material for: Genetic Determinants of Cell Size at Birth and Their Impact on Cell Cycle Progression in Saccharomyces cerevisiae
Source: G3 (Bethesda). 2013 Sep 1;3(9):1525–30. doi: 10.1534/g3.113.007062 (PMC3755912; doi:10.1534/g3.113.007062)
Supplement: Supporting Information [file supp_g3.113.007062_FigureS1.pdf]

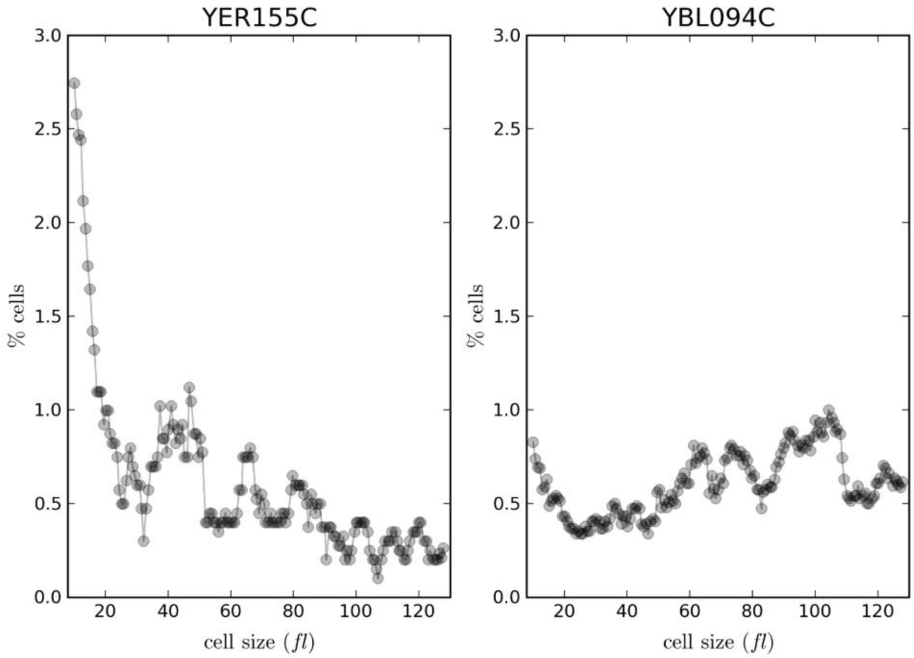

Figure S1 Examples of censored cell size distributions. These mutants' distributions were too irregular to define a daughter cell range, and they were removed from the mutant set analyzed in this paper.
