# Supplementary material for: Genetic Determinants of Cell Size at Birth and Their Impact on Cell Cycle Progression in Saccharomyces cerevisiae
Source: G3 (Bethesda). 2013 Sep 1;3(9):1525–30. doi: 10.1534/g3.113.007062 (PMC3755912; doi:10.1534/g3.113.007062)
Supplement: Supporting Information [file supp_g3.113.007062_FigureS2.pdf]

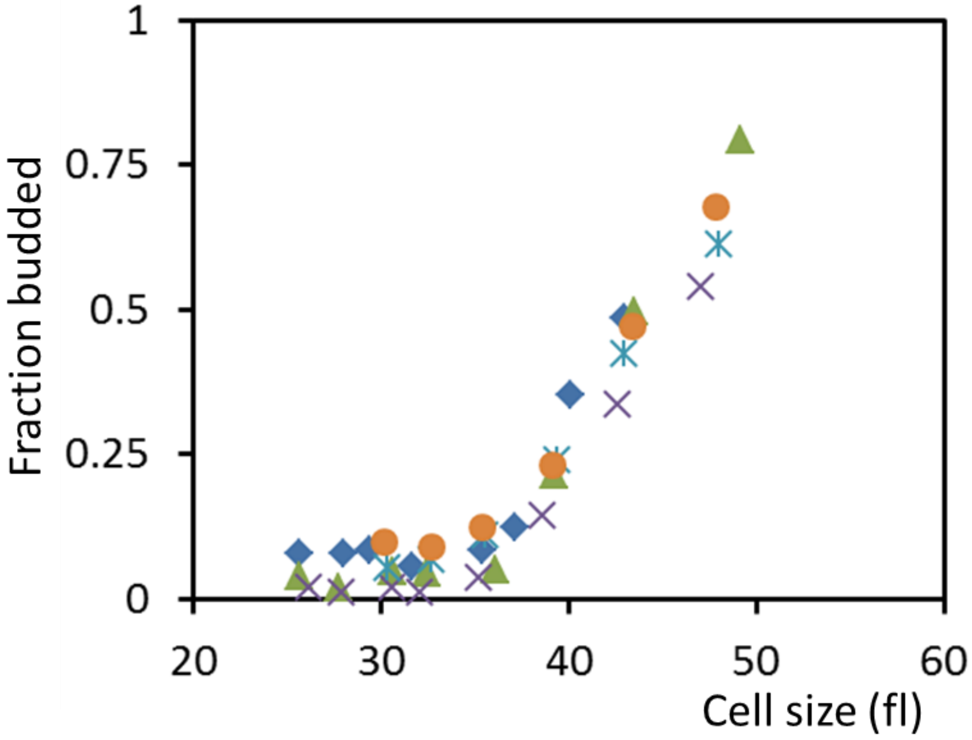

Figure S2 Budding as a function of size for strain W303a in synthetic complete medium. Cells were cultured and elutriated on five separate experiments. Every 20 min the fraction of budded cells was measured microscopically (y-axis), and plotted as a function of the mean cell size of the population (x-axis).
