# Supplementary material for: Genetic Determinants of Cell Size at Birth and Their Impact on Cell Cycle Progression in Saccharomyces cerevisiae
Source: G3 (Bethesda). 2013 Sep 1;3(9):1525–30. doi: 10.1534/g3.113.007062 (PMC3755912; doi:10.1534/g3.113.007062)
Supplement: Supporting Information [file supp_g3.113.007062_FigureS3.pdf]

A

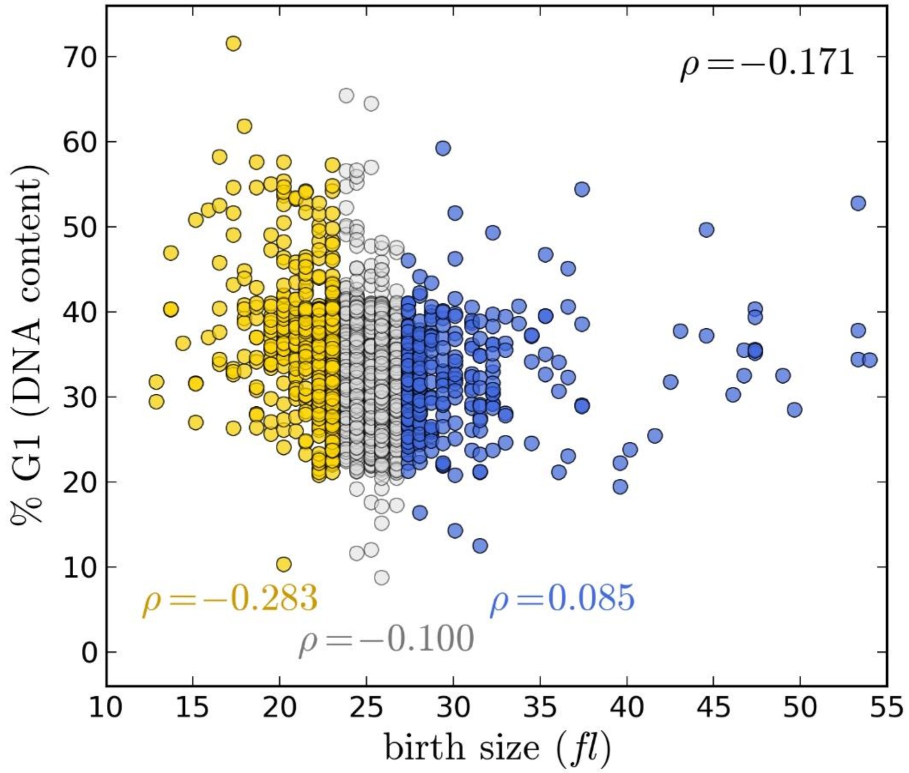

B

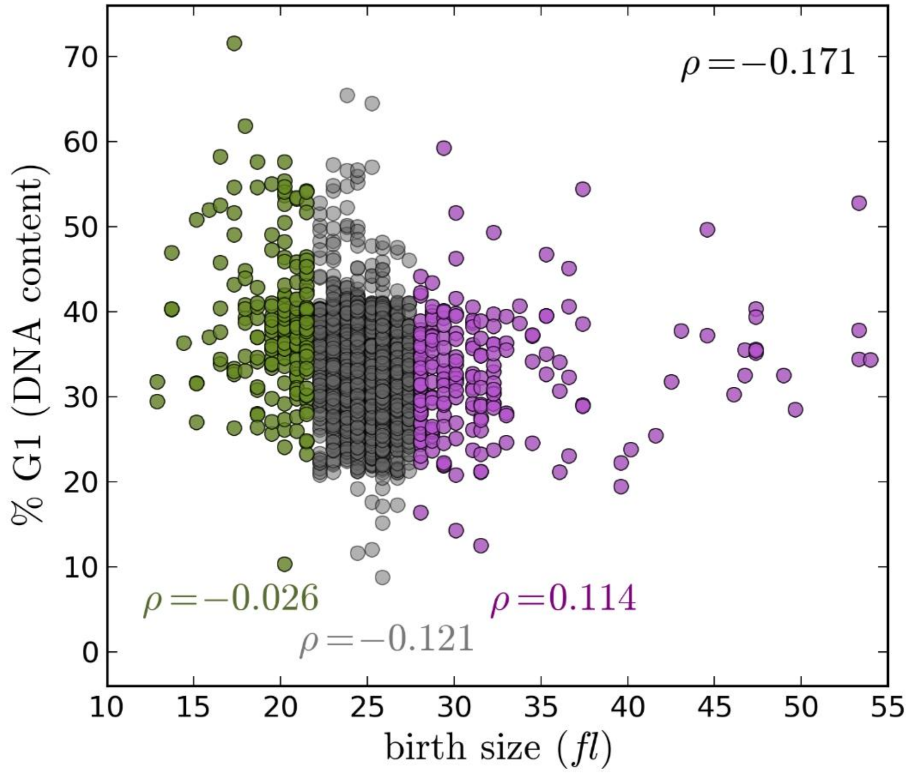

Figure S3 Correlation of birth size with DNA content. %G1 DNA content values were obtained from (Hoose *et al.* 2012). In A and B, the data were colored and displayed as in Figure 3, using again the 20% cutoff for birth size estimates (see Materials and Methods).
