# Supplementary material for: Genetic Determinants of Cell Size at Birth and Their Impact on Cell Cycle Progression in Saccharomyces cerevisiae
Source: G3 (Bethesda). 2013 Sep 1;3(9):1525–30. doi: 10.1534/g3.113.007062 (PMC3755912; doi:10.1534/g3.113.007062)
Supplement: Supporting Information [file supp_g3.113.007062_TableS1.pdf]

Table S1 Statistics of comparisons between different categories of birth size mutants and their corresponding %G1 DNA content

Comparisons	Test	p-value
Small vs. normal birth size (see Fig. 5A)	t-test	1.3192E-37
	Mann-Whitney	5.9898E-26
Large vs. normal birth size (see Fig. 5A)	t-test	0.48
	Mann-Whitney	0.22
Small vs. normal birth size (see Fig. 5B)	t-test	2. 7059E-55
	Mann-Whitney	8. 1026E-33
Large vs. normal birth size (see Fig. 5B)	t-test	0.53
	Mann-Whitney	0.24
